# Exploring Potential Benefits of Accumulated Multicomponent-Training in Non-Active Older Adults: From Physical Fitness to Mental Health

**DOI:** 10.3390/ijerph18189645

**Published:** 2021-09-13

**Authors:** Pablo Monteagudo, Ana Cordellat, Ainoa Roldán, Mari Carmen Gómez-Cabrera, Caterina Pesce, Cristina Blasco-Lafarga

**Affiliations:** 1 Sport Performance and Physical Fitness Research Group (UIRFIDE), University of Valencia, 46010 Valencia, Spain; Ana.Cordellat@uv.es (A.C.); Ainoa.Roldan@uv.es (A.R.); 2 Department of Education and Specific Didactics, Jaume I University, 12071 Castellon, Spain; 3 Physical Education and Sports Department, University of Valencia, 46010 Valencia, Spain; 4 Freshage Research Group, Department of Physiology, Faculty of Medicine, University of Valencia, CIBERFES, Fundación Investigación Hospital Clínico Universitario/INCLIVA, 46010 Valencia, Spain; Carmen.Gomez@uv.es; 5 Department of Movement, Human and Health Sciences, Foro Italico University, 00135 Rome, Italy; caterina.pesce@uniroma4.it

**Keywords:** active aging, elderly, executive function, instrumental activities of daily life, sedentary behavior, strength, physical exercise, walking speed, wellness

## Abstract

The present study aimed to analyze the impact of a multicomponent training (MCT) program in a group of non-active older adults, comparing two different dose distributions. Twenty-four individuals, assigned to two groups, completed 15 weeks of MCT (2 days/week). The continuous group (CMCT; n = 14, 9 females; 71.07 ± 5.09 years) trained for 60 min/session in the morning. The accumulated group (AMCT; n = 10, 5 females; 72.70 ± 3.59 years) performed the same exercises, volume, and intensity, but the training was distributed twice per day (30 min in the morning; 30 more in the afternoon). Bonferroni post hoc comparisons revealed significant (*p* < 0.001) and similar large improvements in both groups in lower limb strength (five times sit-to-stand test: CMCT, 12.55 ± 2.83 vs. 9.44 ± 1.72 s; AMCT, 10.37 ± 2.35 vs. 7.46 ± 1.75 s). In addition, there were large gains in preferred walking speed and instrumental daily life activities, which were higher for CMCT and AMCT, respectively (in this order: 1.00 ± 0.18 vs. 1.44 ± 0.26 m/s and 1.09 ± 0.80 vs. 1.58 ± 0.18 m/s; 33.07 ± 2.88 vs. 36.57 ± 1.65 points and 32.80 ± 1.93 vs. 36.80 ± 0.92 points); improvements in cardiorespiratory fitness, now moderate for CMCT (474.14 ± 93.60 vs. 529.64 ± 82.76 m) and large for AMCT (515.10 ± 20.24 vs. 589.60 ± 40.38 m); and medium and similar enhancements in agility in both groups (TUG test: CMCT: 7.49 ± 1.11 vs. 6.77 ± 1.16 s; AMCT: 6.84 ± 1.01 vs. 6.18 ± 0.62 s). None of the protocols had an impact on the executive function, whereas health-related quality of life showed a trend to significance in the whole sample only (EQindex overall sample, *p* = 0.062; *d* = 0.48 CMCT; *d* = 0.34 AMCT). Regardless of the type of dose distribution, starting multicomponent training improves physical function in non-active older adults, but does not improve cognitive function at mid-term. Because both forms of MCT showed similar compliance, slightly positive differences in accumulated strategies may indicate some benefits related to breaking afternoon sedentary behaviors, which deserves further research in longer and larger interventions. The mixed nature of MCT suggests accumulative group interventions may be a promising approach to address sedentary aging.

## 1. Introduction

Western high-income countries face the challenge of deleterious sedentary ageing, which is also increasing in emergent economies [1,2]. Europe is likely to face higher costs of long-term care and healthcare in coming years [1]. To counteract this global pandemic, international policies and governments focus on the reduction of physical inactivity and/or sedentary behaviours [3,4,5,6]. These two related, expensive issues lead to physical and mental dysfunction [4], increased frailty and loss of independence [6], hypertension [7], immunosenescence [8], inflammaging [9], and higher rates of comorbidity and overall mortality [5]. In this scenario, developing the best strategies (i.e., cost-efficient and cost-effective interventions) is now of paramount importance [3,10]. This highlights the need not only for a psycho-physiological individual approach, but also for a comprehensive global/environmental analysis [11].

The physical and psychological benefits of regular physical activity (PA) for older adults (OA) have been widely documented [8,12,13,14,15]. These benefits broadly encompass not only physical, but also mental and psychosocial health [14,16]. However, 31% of the world’s population may not currently meet the recommended levels of PA [5,17], despite the efforts of different institutions and governments to promote active lifestyles [10,18,19]. The prevalence of physical inactivity in older Europeans (≥55 years) ranges from 5% to 29% [20], confirming the need to promote physical activity and active aging. Improving health through supervised and structured programs of physical exercise in older adults is thus an international health objective and a public health challenge [2,3,10,21].

To pursue the aims of holistic health development and maintenance, both PA dose and quality must be considered and appropriately designed [22]. Training should be structured in ways that ensure that overall increments in physical active time and physical fitness [23] translate into preserving functional mobility [13] and good mental and psychosocial health [16]. A relevant aspect of PA training that may contribute to holistic health development is the extent to which it also challenges high-level cognition, such as executive function, relevant to health, wealth, safety, and success in multiple life domains [24]. In this regard, motor-cognitive dual task training with challenge progression appears to be a suitable approach to the design of PA interventions that benefit both functional mobility and executive function in older adults [25,26].

Moreover, it is not only important to practice physical exercise—designed to meet multiple physical and non-physical health needs—in a systematic or scheduled way, but also to reduce sedentary behaviors throughout the day, because prolonged bouts of sedentary behavior may damage metabolic health and physical function, independently of moderately vigorous physical activity [7,27,28,29]. It is also of outermost importance to understand usual sedentary behavior patterns in older adults to implement effective strategies. Some authors have found that afternoon and early evenings time slots are periods with high levels of sedentary behavior [4,30], which should be regularly interrupted to avoid the pathological consequences of excessive sitting [4,7,29]. Moreover, the so-called accumulative exercise bouts (of at least 10 min, distributing exercise training in the morning and in the afternoon) are indicated to be an effective strategy to counteract sedentary time in less-active timeslots [7,15,31].

In this context, accumulative exercise walking programs have shown improvements in sedentary seniors’ physical function [32,33], in addition to improvements in body composition similar to those found in the continuous approach [34]. Accumulative exercise has also shown to be as effective as longer bouts of exercise in improving plasma lipid profiles, fasting plasma insulin levels, blood pressure, and body composition [34,35,36]. Moreover, we previously found beneficial effects on body composition, regardless of the use of a continuous or accumulated strategy, following a multicomponent training (MCT) program [34]. However, although the accumulated MCT approach to training emerged in the past decade as an effective method for stimulating the overall functioning capacity of older adults, there are a lack of studies regarding the effect of accumulated MCT on this cohort’s physical function [37]. MCT, defined as a physical exercise program that contains aerobic and resistance exercises, balance, motor control, and mobility stimulation [38], is focused on comprehensive responses in the subject in addition to an overall systemic activation [39]. Consequently, these exercise interventions improve cardiorespiratory fitness, neuromuscular function, health-related quality of life, and body composition [40,41,42,43]. Furthermore, the addition of cognitive demands to physical exercise constitutes a better strategy to improve not only physical, but also cognitive outcomes, compared to training programs with an isolated physical capacity [44,45]. When performed in a group, MCT also promotes socialization and adherence to exercise [43,44].

Therefore, the present study aimed to analyze the impact of a MCT program on physical function, executive function, and health-related quality of life in a group of non-active older adults comparing two different dose distributions (accumulative versus continuous). We evaluated if a particular type of the aforementioned multicomponent program, which has shown significant improvements in physical function and mental health [34,39,44,46,47,48,49], is more or less beneficial when carried out accumulatively throughout the day. We hypothesized that both strategies will effectively improve physical and mental health in non-active older adults starting regular training. However, we explored whether there are selectively larger effects of MCT on specific facets of physical and cognitive function and quality of life if the training is performed in a continuous or accumulated fashion.

## 2. Materials and Methods

### 2.1. Participants

From December 2016 to January 2017, twenty-seven participants were recruited from the Health Care Centre of Buñol, a rural environment near Valencia, Spain. Recruitment was based on a medical derivation regarding the following criteria:

Inclusion criteria: age ≥ 60 years; be non-active (no participation in a regular exercise program or intentional activities beyond normal daily habits within the previous 4 months); reporting a gait speed higher than 0.6 m/s; and sufficiently physically and mentally fit to able to participate in a regular exercise program according to the medical referral.

Exclusion criteria: Presentation of any disorder that prevented the participant from being able to complete a training program; to have an adherence lower than 75% to the training sessions; and missing 4 or more consecutive training sessions.

These criteria were first discussed with the medical staff (doctors and nurses) who conducted the screening interviews at the hospital. For instance, they were informed that participants would need a minimum cognitive capacity to face the dual-task constraints in the program, of at least the traditional 24 point cut-off in the Mini Mental State Examination [50,51]. After medical referral, a second screening session was conducted in the hospital, in which one sport sciences researcher interviewed the participants to ensure the fulfillment of the criteria. All individuals were specifically asked about their participation in any regular supervised physical activity during the previous four months, including walking, dancing, or any other exercise training, in addition to any rehabilitation session/program. Participants also were informed about the fact that they were not able to participate in other supervised exercise programs during the intervention.

Older individuals previously provided written informed consent to participate in this study, which was approved by the ethics committee of the University of Valencia (H1484058781638). One individual failed the inclusion criteria in the first screening; thus, twenty-six participants were homogeneously stratified into 2 groups in terms of age, sex, body mass index (BMI), and gait speed over a distance of 6 m. Two additional participants dropped out during the intervention for reasons not related to the study; thus, twenty-four older adults comprised the final sample included in the statistical analysis.

### 2.2. Research Design

This quasi-experimental and longitudinal study was carried out with a pre-post design of one factor: the dose distribution of the MCT program. The continuous MCT (CMCT) group trained for 60 min/session, always in the morning, whereas the accumulated MCT (AMCT) group performed the same duration, types, and sequences of exercises, but distributed twice per day, performing 30 min in the morning and 30 min in the afternoon, with at least 5 h separating each exercise bout.

Medical staff and sport sciences researchers collaborated in the initial testing sessions during February 2017, for demographic, biological, physical, and functional assessment, in addition to assessment of executive function, and questionnaires relating to health-related quality of life and instrumental activities of daily living (IADL). The MCT programs were performed in the same local public sport facilities for 15 weeks, and were supervised and tailored on a daily basis by 2 sport sciences graduates. Participants were re-evaluated during June 2017 by reproducing the same protocols.

### 2.3. The EFAM-UV© Multicomponent Training Program

The exercise intervention consisted of 30 sessions (twice/week over 15 consecutive weeks) for the CMCT group, and 60 sessions (twice/week but distributed in two timeslots in the same day over 15 consecutive weeks) for the AMCT group. The MCT followed the EFAM-UV© methodology [52] (Spanish acronym for *Entrenamiento Funcional para Adultos Mayores*). As previously described [34,39,44,46], this MCT is based on gait retraining and improving postural control with constraints and enriched environments to increase the cognitive demands. Neuromuscular and cardiovascular proposals under the dual-tasking approach were combined to exert systemic and comprehensive responses, mainly according to this structure of session:

(1) 10 to 15 min neuromuscular activation, based on gait training, plus postural control exercises, increasing the cognitive executive constraints according to the individual capacities.
(2) 15 to 20 min of neuromuscular development strength plus balance exercises (exercises with elastic bands and dumbbells on alternating days, increasing their demands on motor control).
(3) 15 to 20 min of bioenergetics (by means of gait training sequences, rhythm exercises, or functional motor skills) on different days, depending on the periodized objectives.
(4) 5 to 10 min of cool down with playful and social tasks (tailoring the social interaction tasks in a way that included executive function challenges whenever possible, because both social interaction and executive function share common important mechanisms that are benefited by exercise [53]).

**Figure 1:**
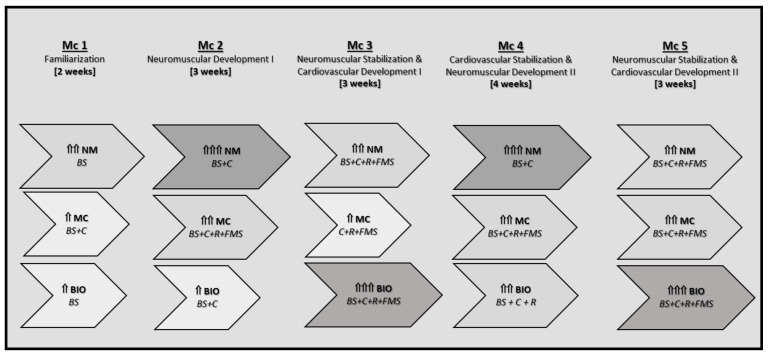
The EFAM-UV© periodization. Its six dimensions comprise two main basic skills (BS: gait training and postural control) plus two complementary skills (C: manipulative and cognitive) at a first level. In a second level of the EFAM-UV© taxonomy there are the rhythm tasks (R) and functional motor skills (FMS). Horizontal arrows represent the strain in each domain and the contents’ orientation (NM: neuromuscular; MC: motor control; BIO: bioenergetics). The length and vertical small arrows (and color hues) reflect the prevalence and importance of each motor domain (see Ref. [44] for a deep description of this MCP).

As mentioned above, this multicomponent neuromotor exercise training methodology has shown improvements in different populations of older adults [34,39,44,46,47,48,54]. A medium duration (i.e., 15 weeks) may be enough [55] to result in improvements in this population. Accordingly with the guidelines of the EFAM-UV©, the intervention was tailored and periodized from neuromuscular to bioenergetics demands, in which executive function was a permanent target, by adjusting the main and typical mesocycles (Mc) of the program to the 15 week macrocycle performed in this study (Figure 1). As previously described [44], exercise progressions by means of tailored physical conditioning maps allows metabolic demands to be increased without reducing, or even augmenting, executive function requirements.

### 2.4. Outcomes

To evaluate physical function, the six-minute walk test (6MWT) was assessed for cardiorespiratory fitness; grip strength (GS) and the five times sit-to-stand test (FTSST) were considered for upper- and lower-limb strength, respectively; and the timed up and go test (TUG) was applied for agility and dynamic balance. Preferred walking speed (PWS), or “self-paced walking speed” was also assessed by means of two electric photocells and the Chronojump Software (Velleman PEM10D photocell, Chronojump Bosco System, response time 5–100 ms).

A battery of questionnaires composed of the EQ-5D-5L [56], the VIDA questionnaire [57], and the Stroop Color and Word Test [58] was included to assess health-related quality of life, IADL, and executive function, respectively. More specifically, the EQindex (utility index) and the EQVAS (visual analogue scale) was used from the EQ-5D-5L, and the interference (IN) was calculated from the Stroop Test as a representative value of the executive function [59].

Finally, other biological parameters, such as age, sex, weight, height, BMI, blood pressure, oxygen saturation (SpO2), and heart rate (HR), were also collected to characterize the sample. For more details about both the protocol of each test and the instruments of measurement used, refer to these studies [33,34,41,60].

### 2.5. Statistical Analysis

The analysis of the data was performed with the SPSS statistics package version 23 (IBM SPSS Statistics for Windows, Chicago, IL, USA). After testing for normality (Shapiro–Wilks), Student’s t test, or the Mann–Whitney U test (SpO2 and HR) were first applied for baseline group comparisons. A repeated measures ANOVA was then conducted to analyze changes in health-related quality of life and functional measures, considering the main effect of the intervention (pre-post overall comparison) and the interaction between type × dose distribution (CMCT vs. AMCT). Within-subjects effects tests at the first level, followed by Bonferroni post hoc tests, were performed with statistical significance set at the level of *p* ˂ 0.05. Subsequently, to homogenize and analyze these changes, the effect size (ES) was calculated by means of Cohen’s d, where the effects were considered to be small (*d* = 0.20–0.49), medium (*d* = 0.50–0.79), or large (*d* ≥ 0.80) according to Cohen [42]. Descriptive statistics are expressed as mean ± standard deviation (SD). Changes in functional and health-related quality of life variables were further expressed as percentage of change (calculated by means of the formula (post-score–pre-score)/pre-score x 100). Student’s t test or the Mann–Whitney U test were applied for group comparisons within deltas. Individual variables were checked for homogeneity of variance using Levene’s test.

## 3. Results

Table 1 includes the main physical characteristics of the two groups, which were homogenous at the baseline. They showed no statistically significant differences in terms of age, sex, weight, height, BMI, SBP, DBP, SpO2, and HR. Functional and health-related quality of life outcomes were not different at baseline (*p* > 0.05). Participants completed the intervention with an adherence rate of 88.82% (89.52% for CMCT vs. 87.83% for AMCT).

Regarding the main effect of the “intervention” in the whole sample (n = 24) (Table 2), the within-subjects effects test of the repeated measures ANOVA showed a significant main effect (*p* ˂ 0.05) in the functional outcomes. Improvements were significant in lower limb strength (FTSST: 11.64 ± 2.81 vs. 8.62 ± 1.96 s), cardiorespiratory fitness (6MWT: 491.21 ± 74.42 vs. 554.62 ± 73.63 m), preferred walking speed (PWS: 1.04 ± 0.15 vs. 1.50 ± 0.24 m/s), agility (TUG: 7.22 ± 1.10 vs. 6.53 ± 1.00 s), and in perceived autonomy (IADL: 32.96 ± 2.48 vs. 36.67 ± 1.37). In addition, health-related quality of life (EQindex) showed a trend to significance (0.85 ± 0.14 vs. 0.90 ± 0.10). No significant changes were found for body mass index, upper limb strength, executive function, or EQVAS.

There was no effect of the interaction of “intervention × dose distribution” for any variable (Table 2).

A post hoc analysis with the G*Power software (v 3.1.9.4) revealed that a sample size of 24 subjects (alpha = 0.05) gave a power of 0.96 for an ES mean of 0.4 considering the ANOVA repeated measures, within-between interactions. The power dropped considerably when considering the small effects of the interaction, indicating the need of a larger sample size in the two-group analysis.

Further Bonferroni analyses of pre-post differences (Table 3) showed significant improvements for both strategies on FTSST, 6MWT, PWS, TUG, and IADL, with big and moderate ESs. FTSST showed similar big ESs in both groups (d ≈ 1.35), whereas IADL showed big ESs with a slight and superior difference for the AMCT group. Large gains were also found in PWS, which were higher for CMCT than AMCT. Improvements in 6MWT were moderate for CMCT (*d* = 0.63) and very large for AMCT (*d* = 2.33). Both groups showed similar medium effect sizes for TUG.

Although health-related quality of life outcomes showed improvements with small ESs, these were not at the significance level. No statistically significant variations were detected in any group for IN, BMI, and GS.

**Figure 2:**
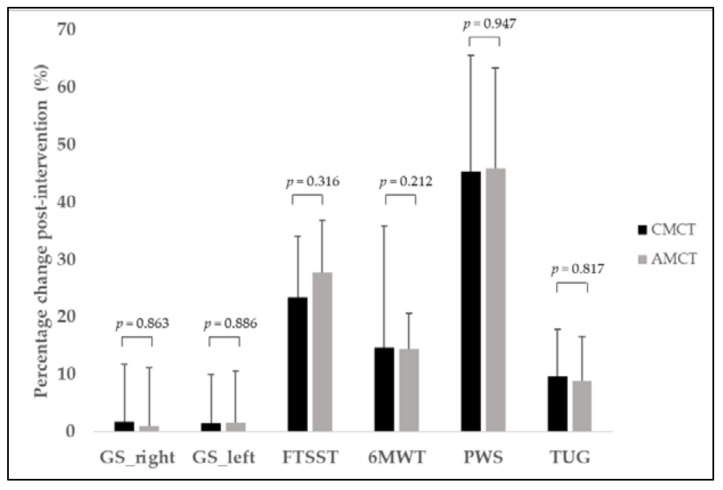
Percentage change in physical function tests following CMCT (solid bar) and AMCT (gray bar). GS indicates grip strength; FTSST, lower limb strength; 6MWT, 6 min walk test; PWS, preferred walking speed; TUG, timed up and go; CMCT, continuous multicomponent training; AMCT, accumulated multicomponent training. For GS, 6MWT, and PWS, the greater the percentage change, the greater the improvement. For FTSST and TUG, the lower the percentage change, the greater the improvement.

**Figure 3:**
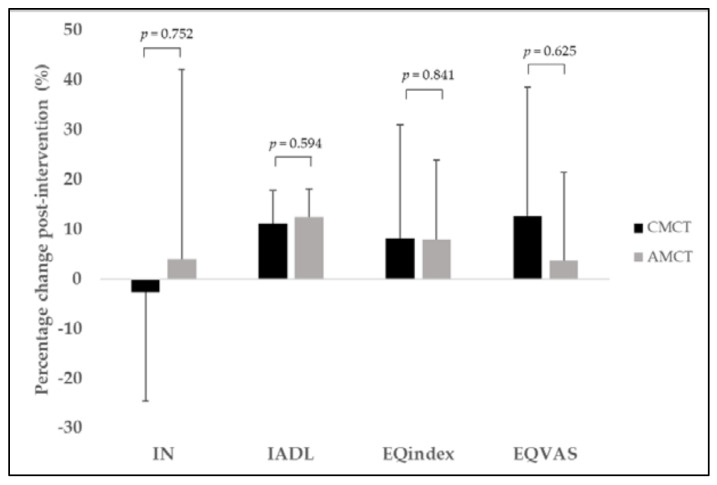
Percentage change in executive function, Instrumental Activities of Daily Living, and health-related quality of life measurements following CMCT (solid bar) and AMCT (gray bar). IN, inhibition; IADL, Instrumental Activities of Daily Living; CMCT, continuous multicomponent training; AMCT, accumulated multicomponent training. For all variables, the greater the percentage change, the greater the improvement.

The percentage changes in the main outcomes measured in our study are reported in Figure 2 (physical function parameters) and Figure 3 (executive function, IADL, and health-related quality of life). We did not find statistically significant differences between CMCT and AMCT in any of the measurements performed.

## 4. Discussion

The aim of our study was to compare the effect of accumulated and continuous MCT programs on sedentary older adults, to analyze any potential benefits related to the dose distribution after 15 weeks of MCT. To the best of our knowledge, this is the first study to compare a dose strategy in this specific type of periodized exercise to fight sedentary aging. Based on the literature, we expected a general beneficial effect of MCT [34,37,38,39,44,46,61], and hypothesized selectively larger effects on specific facets of physical and cognitive function and quality of life, depending on the dose strategy. As a main finding, our results confirm the benefits of both interventions on physical function, with effect size differences on cardiorespiratory fitness and perceived autonomy. However, we found no improvements in grip strength or interference, and a trend to significance was only found in quality-of-life perception when considering the effect of the intervention in the whole sample (n = 24).

It is well known that regular physical activity evokes relevant benefits in sedentary older adults, indicating that even a small increase in physical activity may produce large gains [39,62]. Among different types of physical exercise, MCT has been recommended in older adults because it yields improvements in several components of physical fitness, rather than in a specific component, emphasizing that all components are affected by aging [61,63], and are also essential for maintaining functional independence [63]. Not only is the type of exercise important, but also the ability of these programs to bring about changes with a low economic cost, and the attractiveness of the programs to the elderly to create adherence [3,4,11,21]. It is essential to tailor the physical exercise interventions to individuals to support the maintenance of physically active lifestyles [11,64,65]. In this sense, the EFAM-UV© program is a good proposal because it shows improvements after 15 weeks of training on only two days per week, in addition to generating excellent adherence in both dose strategies (89.52% vs. 87.83% for CMCT and AMCT, respectively), similar to previous interventions [44,66]. The multimodal and blocked structure of the EFAM-UV© methodology helped to achieve the large and similar adherence in the two strategies, because exercises and progressions were easily matched and distributed, regardless of the use of the continuous or accumulative sessions, without giving older adults the feeling of monotony or repetition. This is relevant because this is the first work that compares the continuous and accumulative strategies in a multicomponent program.

For this reason, it is difficult to compare our results with previous research. Moreover, the few studies that have compared the effect of continuous and accumulated supervised exercise bouts have only focused on cardiorespiratory fitness. For example, Magutah et al. [67] found that accumulated moderate intensity jogging bouts of <10 min confer similar-to-better cardiovascular improvements compared with current recommendations among sedentary adults over 50. Consistent with these studies, our accumulated strategy showed greater improvements in cardiorespiratory fitness, namely, the large ES (*d* = 2.3), unlike the medium ES obtained in the continuous group (*d* = 0.6; n = 14). Most importantly, a similar response was found for perceived autonomy (*d* = 2.6 vs. *d* = 1.5 in accumulated vs. continuous training, respectively), emphasizing the closer association between IADL and bioenergetics [68], in addition to physical function in general [68,69]. We found no between-groups differences in the pre-to-post change in cardiorespiratory fitness and perceived autonomy, probably due to the large heterogeneity in the older adults’ response to exercise (Figure 2 and Figure 3), the slight sample differences in the final size of the two groups, and the reduced statistical power when separately considering the two dose distribution groups.

These benefits for the accumulated group (i.e., larger ES in cardiorespiratory fitness and perceived autonomy; n = 10) can be ascribed not exclusively to the training itself, but also to the framework conditions of training in two separate sessions instead of one continuous session. In the accumulated strategy, participants were required to travel to the local sports facilities twice per day (often walking), which may have translated into higher cardiovascular work. As a further positive side effect, this distribution may have reduced the sedentary time in the afternoon, thus interrupting excessive sitting and favoring activity in this time slot and allowing older adults to cope with other tasks and to be more rested. Similarly, Jindo et al. [70] compared the effects of a multicomponent program in two groups, in which participants in one of the groups wore pedometers during the intervention. Results from this study showed larger improvements in physical function for the pedometer group, suggesting that pedometers invited the participants to walk outside in addition to the supervised MCT sessions.

Further research with larger samples should confirm this relevant point. Physical activity and sitting time may have both joint and independent effects, and are not merely the extremes of a continuum [4,71]. Indeed, a health-enhancing dose of PA cannot completely prevent the negative effects of prolonged sitting time [4]. Greater amounts of more vigorous physical activity effectively eliminate the association of sitting time with all-cause mortality and cardiovascular mortality risk. However, reducing the sitting time has been shown to be an important strategy, ancillary to increasing physical activity in physically inactive populations [71]. Thus, it appears that the selection of the type, intensity, and frequency of the physical activity to effectively counteract the detrimental effects of prolonged sitting may differ according to subjects’ characteristics, and particularly their habitual physical activity level [29]. Accumulated MCT may be a potential option when starting a physical exercise intervention in sedentary older adults.

Regarding the neuromuscular field, PWS showed a percentage change of 44% in both strategies. However, in contrast to the differential group effects observed for cardiovascular fitness, the continuous group (n = 14) reported a greater ES (*d* = 1.97 vs. *d* = 0.84). Some authors have suggested that a lower energy cost of walking may allow a higher habitual walking speed [72]; however, 60 continuous minutes of the EFAM-UV© program may ensure more attention is focused on the gait control and gait demands compared to distributing this dose, despite less ES in cardiorespiratory fitness.

Our results also confirm that no dose strategy succeeded in improving upper limb strength, despite the similar improvements in dynamic balance (TUG) and lower limb strength (FTSST), independently of the protocol. It is important in older adults to improve the latter, as shown in previous interventions [44,73], which achieved improvements of between 10% and 30% in a similar manner and with a large and medium ES, respectively, because these outcomes are related to preventing the risk of falls [74,75]. It is likely that, because the EFAM-UV© program is structured and periodized, it favored improvements in lower limb strength regardless of the distribution strategy, because the time of motor involvement was the same. However, it is also important to highlight that the EFAM-UV© program is based on gait control and re-education, and does not focus on upper limb strength. In addition, the manipulative skills are more focused on the maintenance of postural control and skill (transport, grab, release, pass, bounce, etc.) than on the grip strength itself. Focusing on core and lower limb muscle strength and functionality, together with executive function and socialization, may be a limitation for hand grip improvements, regardless of the dose distribution. However, the fact that the MCT program does not show significant differences in terms of a worsening of the grip strength indicates a certain positive effect of both strategies on the strength of the upper limb compared to the deterioration expected due to the aging process.

Related to mental health, our results indicate that dose distribution is not a determinant of either health-related quality of life or the cognitive effects associated with MCT in sedentary adults.

Unfortunately, we did not find changes in inhibition (executive function) after the intervention in either of the two groups. It is possible that the cognitive stimulation embedded in the MCT was not sufficiently specific to challenge the facet of inhibition (interference control) that we tested. Indeed, there is a large degree of consistency in the literature regarding the lack of transfer of the effects from a specifically trained executive function to others [76,77]. Considering the age-related worsening of inhibition in the absence of a specific stimulation [78], maintaining executive function levels can again be considered a beneficial effect in this kind of population [79].

In this regard, Jefferis et al. [80] argue that, in older men, the accumulation of physical activity in bouts ≥10 min does not appear to be more important than the total volume of activity. Similarly, Peven et al. [81] found evidence that the total volume of moderate-vigorous physical activity is more influential on executive function than the breakdown of longer (i.e., ≥10 min) or shorter (i.e., <10 min) bouts of activity, indicating the importance of exercise intensity and fatigue associated with total volume. In addition, it is notable that our research group recently found that cognitive function may improve after a period of training cessation [44], which may be explained by slower and delayed development/changes compared to physical function [45,82].

By comparison, with regard to health-related quality of life, we found a trend to significance in EQindex when considering the whole sample (n = 24), and positive but not significant changes when observing both groups separately for EQVAS and EQindex. Health-related quality of life is an important outcome in older adults because it provides a complete understanding of their general care [64]. The trend to improvement found in these outcomes may be related to changes in physical function, due to the strong association that usually exists between these variables [60,83]. We recently found that physical function may influence health-related quality of life more than other factors (such as BMI, age, or the cognitive function), at least in a healthy and homogeneous population of older adults [60]. Moreover, we adopted a group approach, in which social interaction tasks between the participants were performed [43,44], because previous studies demonstrated that social interaction and exercising with others is clearly more significant, and leads to better mental health, than exercising alone [84]. Finally, the friendly and supportive attitude of the instructors, who modified the intervention content to suit individual preferences and needs, may also have helped health-related quality of life results.

Importantly, this study was subject to a number of limitations. First, we did not quantify physical activity levels using accelerometry or complementary questionnaires, either pre-intervention, which could have confirmed the sedentary level of the sample, or post-intervention, which could have provided information about possible changes in sedentary patterns. Secondly, the small size of the sample (n = 24), and the fact that we did not include a control group who did not exercise, may have implications for the generalizability of the study results. In addition, we did not collect information on key confounding factors, such as smoking and drinking habits, and other socio/economic indicators. In addition, it may have been more appropriate to introduce exclusion criteria related to cognitive and mental capacities, and to assess executive function with a motor dual task (or even a motor-cognitive dual task), rather than using a cognitive dual task (Stroop Test), because it may have better reflected the possible specific effect of the EFAM-UV© program on this outcome. Finally, the lack of previous experience in applying this mixed approach, which was initiated in a hospital and developed in local sport facilities, to comprehensive health interventions may have required more coordination between multidisciplinary professionals (i.e., medical services and sport sciences professionals). Notably, a measurement over time would have been interesting to observe if adherence to exercise (and therefore regular physical activity) was maintained after the intervention, together with any delayed benefit for mental health. Future studies should address the role of longer interventions on these variables and this possible delayed benefit after detraining, which may be related to better socialization, perceived social support, self-efficacy, and perceived safety [85], thus positively influencing the older adults’ health-related quality of life. In addition, it would have been interesting to assess the levels of physical activity in addition regular exercise in our study, to know if the intervention modified patterns of physical activity outside the exercise sessions (between groups and/or within groups). In summary, despite the lack of sufficient evidence to determine the most effective means to change sedentary behavior in older adults, multicomponent approaches combining behavioral changes and physical exercise may be among the most suitable [86]. From this comprehensive perspective, the strategy implemented in this intervention may be of significant interest to counteract sedentary aging. Our results are promising and confirm the need to further the understanding of the effects of tailored and supervised bouts of MCT programs on functional and mental health outcomes in the older population, at least as an early first strategy, which was shown to be low cost and effective for sedentary individuals.

## 5. Conclusions and Practical Applications

To the best of our knowledge, this is the first study to compare the benefits of accumulating or concentrating the dose of a MCT on physical function and mental health in non-active older individuals. Our results confirm that, regardless of the dose strategy, 15 weeks of well-tailored and supervised MCT are enough to provide similar benefits on neuromuscular facets of physical function, such as balance and lower-limb strength. Despite important study limitations, such as the reduction of the sample size in the between-group comparisons, larger effect sizes on cardiorespiratory fitness, and perceived autonomy in the accumulative strategy, the results indicate promising benefits which deserve further research.

However, the perceived quality of life tended to improve only when considering the overall sample, and neither of the two tested strategies was able to improve executive function (inhibition), thus limiting the benefits of this type of MCT on mental health, regardless of the dose, at least after a medium-term intervention.

Current health promotion policies suggest that we must progressively move towards a social comprehensive model focused on overall health. Effectiveness and efficiency of physical exercise interventions may benefit from group multimodal approaches, which deserve further research and require additional funding to be improved. Because the identification of powerful strategies to promote active lifestyles, break sedentary behaviors, and engage sedentary older adults in regular physical activity is urgently needed, early accumulated MCT interventions may be of interest. The promising results of the present study encourage the development of future studies to confirm that accumulative exercise strategies, combining multimodal approaches rather than relying on walking alone, are health and cost effective.

## Figures and Tables

**Table 1:** Physical characteristics at baseline.

	Total, N = 24	CMCT, N = 14	AMCT, N = 10	*p* ^a^
Age	71.75 ± 4.51	71.07 ± 5.09	72.70 ± 3.59	0.395
Weight, kg	72.42 ± 13.86	72.95 ± 12.39	71.67 ± 16.37	0.829
Height, m	1.59 ± 0.09	1.59 ± 0.09	1.59 ± 0.10	0.912
BMI, kg/m^2^	28.12 ± 3.50	28.35 ± 3.46	27.79 ± 3.70	0.708
SBP, mmHg	149.12 ± 19.38	145.29 ± 20.40	154.50 ± 17.43	0.260
DBP, mmHg	82.42 ± 9.89	81.43 ± 10.17	83.80 ± 9.86	0.574
SpO2, %	96.96 ± 1.33	96.93 ± 1.33	97.00 ± 1.41	0.886
HR, bpm	72.58 ± 9.50	71.14 ± 6.83	74.60 ± 12.46	0.391
Sex				
Females, % (n)	58.30 (14)	64.30 (9)	50.00 (5)	0.678
Males, % (n)	41.70 (10)	35.70 (5)	50.00 (5)	

BMI: body mass index; SBP: systolic blood pressure; DBP: diastolic blood pressure; SpO2: arterial oxygen saturation; HR: heart rate. *p* ^a^: Differences between groups using independent *T*-test (age, weight, height, BMI, SBP, DBP, and HR), Mann–Whitney U test (SpO2) or Fisher’s exact test (sex distribution between the two groups).

**Table 2:** Tests of within-subjects effects.

ANOVA Factors	Variable	Type III Sum of Squares	df	Mean Square	F	*p*	Partial Eta Squared
Intervention	BMI	1.01	1	1.01	2.393	0.136	0.098
	GS_right_	0.08	1	0.08	0.02	0.892	0.001
	GS_left_	0.33	1	0.33	0.12	0.735	0.005
	FTSST	105.86	1	105.86	79.00	0.001 *	0.782
	6MWT	49291.67	1	49291.67	52.63	0.001 *	0.705
	PWS	2.55	1	2.55	149.00	0.001 *	0.871
	TUG	5.56	1	5.56	30.34	0.001 *	0.580
	IN	2.91	1	2.91	0.09	0.770	0.004
	IADL	164.06	1	164.06	98.21	0.001 *	0.817
	EQindex	0.03	1	0.03	3.87	**0.062 ^ꝉ^**	0.150
	EQVAS	174.21	1	174.21	1.92	0.180	0.080
Intervention × Dose-distribution	BMI	0.41	1	0.41	0.96	0.337	0.042
	GS_right_	1.07	1	1.07	0.25	0.619	0.011
	GS_left_	0.51	1	0.51	0.18	0.676	0.008
	FTSST	0.11	1	0.11	0.09	0.772	0.004
	6MWT	1052.92	1	1052.92	1.12	0.300	0.049
	PWS	0.01	1	0.01	0.52	0.478	0.023
	TUG	0.01	1	0.01	0.07	0.800	0.003
	IN	24.15	1	24.15	0.73	0.402	0.032
	IADL	0.729	1	0.729	0.44	0.516	0.019
	EQindex	<0.001	1	<0.001	0.01	0.957	<0.001
	EQVAS	49.71	1	49.71	0.55	0.467	0.024

BMI: body mass index; GS: grip strength; FTSST: five times sit-to-stand test; 6MWT: six minute walk test; PWS: preferred walking speed; TUG: timed up and go; IN: interference; IADL: Instrumental Activities of Daily Living; EQindex: Descriptive index of Euroqol; EQVAS: Visual Analogue Scale of Euroqol. Significant differences or trend are highlighted in bold; * *p* ≤ 0.001, ^ꝉ^ *p* ≤ 0.100.

**Table 3:** Measures for continuous (n = 14) and accumulated groups (n = 10).

	Pre-CMCT	Post-CMCT	ES	Pre-AMCT	Post-AMCT	ES
BMI, kg/m^2^	28.35 ± 3.46	28.24 ± 3.27	0.03	27.79 ± 3.70	27.31 ± 2.99	0.14
GS_right_, kg	30.73 ± 10.39	31.11 ± 10.66	0.04	34.46 ± 11.01	34.24 ± 9.57	0.02
GS_left_, kg	28.16 ± 9.36	28.54 ± 9.72	0.04	31.64 ± 10.74	31.60 ± 9.30	0.01
FTSST, s	12.55 ± 2.83	9.44 ± 1.72 **	1.33	10.37 ± 2.35	7.46 ± 1.75 **	1.40
6MWT, m	474.14 ± 93.60	529.64 ± 82.76 **	0.63	515.10 ± 20.24	589.60 ± 40.38 **	2.33
PWS, m/s	1.00 ± 0.18	1.44 ± 0.26 **	1.97	1.09 ± 0.80	1.58 ± 0.18 **	0.84
TUG, s	7.49 ± 1.11	6.77 ± 1.16 **	0.63	6.84 ± 1.01	6.18 ± 0.62 *	0.79
IN	−3.57 ± 8.47	−4.51 ± 7.31	0.12	−8.87 ± 10.28	−6.93 ± 12.89	0.17
IADL	33.07 ± 2.87	36.57 ± 1.65 **	1.50	32.80 ± 1.93	36.80 ± 0.92 **	2.65
EQindex	0.85 ± 0.11	0.90 ± 0.10	0.48	0.86 ± 0.17	0.91 ± 0.12	0.34
EQVAS	74.07 ± 17.97	80.00 ± 10.38	0.40	83.20 ± 10.17	85.00 ± 10.27	0.18

BMI: body mass index; GS: grip strength; FTSST: five times sit-to-stand test; 6MWT: six minute walk test; PWS: preferred walking speed; TUG: timed up and go; IN: interference; IADL: Instrumental Activities of Daily Living; EQindex: Descriptive index of Euroqol; EQVAS: Visual Analogue Scale of Euroqol; ES: Effect Size. ** *p* ≤ 0.001, * *p* ≤ 0.050.

## Data Availability

According to MDPI Research Data Policies, the data presented in this study are available on request from the corresponding authors. The data are not publicly available due to privacy medical reasons.
